# Venous Thromboembolism Prophylaxis in Gynecologic Oncology: A MITO-MaNGO Survey

**DOI:** 10.3390/diagnostics14111159

**Published:** 2024-05-31

**Authors:** Michele Mongelli, Domenica Lorusso, Vanna Zanagnolo, Sandro Pignata, Nicoletta Colombo, Gennaro Cormio

**Affiliations:** 1 Obstetrics and Gynecology Unit, University of Bari “Aldo Moro”, 70124 Bari, Italy; 2 Division of Gynecologic Oncology, Fondazione Policlinico Universitario A. Gemelli, Istituto di Ricovero e Cura a Carattere Scientifico IRCCS, 00168 Rome, Italy; domenica.lorusso@unicatt.it; 3 Department of Gynecologic Oncology, Istituto Europeo di Oncologia, 20141 Milan, Italy; vanna.zanagnolo@ieo.it; 4 Department of Urology and Gynecology, Istituto Nazionale Tumori, Istituto di Ricovero e Cura a Carattere Scientifico IRCCS, Fondazione G. Pascale, 80131 Naples, Italy; s.pignata@istitutotumori.na.it; 5 Gynecologic Oncology Program, European Institute of Oncology, Istituto di Ricovero e Cura a Carattere Scientifico IRCCS, 20139 Milan, Italy; nicoletta.colombo@unimib.it; 6 School of Medicine and Surgery, University of Milan-Bicocca, 20126 Milan, Italy; 7 S.S.D. Ginecologia Oncologica Clinicizzata, IRCCS Istituto Tumori “Giovanni Paolo II”, 70124 Bari, Italy; gennaro.cormio@uniba.it; 8 Department of Interdisciplinary Medicine (DIM), University of Bari “Aldo Moro”, 70124 Bari, Italy

**Keywords:** gynecology oncology, venous thromboembolism, prophylaxis

## Abstract

Cancer-associated thrombosis is the second leading cause of death in cancer patients, and its incidence has been increasing in recent years. This survey was aimed at gathering information regarding the management of thromboembolic prophylaxis within the MITO (Multicenter Italian Trials in Ovarian Cancer)-MaNGO (Mario Negri Gynecologic Oncology) groups. We designed a self-administered, multiple-choice online questionnaire available only for MITO-MaNGO members for one month, starting in May 2022 and ending in June 2022. We processed one response form per center, and 50 responses were analyzed, with most of the respondents (78%) over 40 years old. We found that 82% of them consider thromboembolic prophylaxis in gynecologic oncology to be relevant. In 82% of the centers, a standardized protocol on venous thromboembolism (VTE) prophylaxis is used, which is applied to both patients undergoing surgery and those undergoing chemotherapy. In the remaining 18% of centers, prophylaxis is used exclusively for patients undergoing chemotherapy treatment. Prophylaxis of patients undergoing surgery and chemotherapy treatment is managed in most cases by the surgeon (72%) and oncologist (76%), respectively. Only 26% of respondents use a thromboembolic risk assessment scale, and of these, those used are the Caprini Score (6%), Khorana Score (6%), and Wells Score (2%). The respondents have good knowledge of low-molecular-weight heparin (90%) and average knowledge of dicumarolics (40%), direct oral anticoagulants (DOACs) (68%), and antiplatelet agents (40%). The results of our survey indicate that there is a good awareness of thromboembolic prophylaxis in gynecologic oncology. Nevertheless, it is used less in outpatients than in patients undergoing surgery. Moreover, the thromboembolic risk assessment scores are barely used.

## 1. Introduction

The leading cause of death in middle- and high-income regions of the world is cardiovascular disease, as reported by the World Health Organization (WHO). The incidence of VTE in Europe and the USA is estimated to be 1–2 per 1000 person-years, but varies widely by age, sex, race, and medical conditions [1].

Virchow in 1856 defined the predisposing elements for venous thrombosis as a triad of events: blood stasis, vascular damage, and hypercoagulability [2].

The incidence of cancer-associated thrombosis is increasing worldwide and it is the second leading cause of death in cancer patients [3]. Neoplastic patients have an underlying state of hypercoagulability due to the prothrombotic properties of tumor cells. The risk of VTE is highest in the early period after tumor diagnosis but varies according to tumor type and stage of disease. The risk is higher in patients with thrombocytopenia or hematological diseases such as lymphoma [4]. 

The incidence of VTE in patients with gynecologic malignancy varies between 3% and 25% and is affected by several patient and tumor-related factors. [5]. Specific properties of each type of gynecologic cancer are described as being implicated in a more significant risk of VTE. Ovarian cancer patients have a higher incidence of VTE than other patients with gynecologic malignancies [6]. In the context of cervical cancer, tumor size is related to an increased risk of VTE: a tumor size > 5 cm carries a 9-fold increased risk (10% vs. 1.2%) [7]. Regarding patients with endometrial cancer, the incidence of VTE varies according to tumor histology: grade 3 endometrioid histologies are associated with a higher VTE incidence than low-grade histologies [8].

Nevertheless, surgery itself is a risk factor for VTE, although the cited incidence ranges widely from 0% to 17% [5].

Cancer treatment, chemotherapy, and radiation therapy, in addition to surgery treatment, frequently worsen the incidence of VTE. Furthermore, when there are VTE complications, patients’ mortality increases [9].

Moreover, patients with gynecologic malignancies, mainly in advanced stages, may experience bleeding episodes due to the nature of the tumor itself, which could complicate the management of VTE prophylaxis. A previous systematic review and meta-analysis of patients undergoing gynecologic surgery suggested that while pharmacologic prophylaxis reduces the risk of VTE by about 55%, it carries an increased risk of major postoperative bleeding by a similar percentage [10].

To date, no previous studies have thoroughly investigated the ongoing management of VTE prophylaxis in gynecologic oncology in Italy. In recognition of these data, we led a survey among the two major working groups that include the major Italian centers dealing with gynecologic oncology: the Multicenter Italian Trials in Ovarian Cancer (MITO) and MaNGO (the Mario Negri Gynecologic Oncology group). The Multicenter Italian Trials in Ovarian Cancer (MITO) group is a distinguished consortium of medical professionals and researchers dedicated to advancing knowledge and treatment options for ovarian cancer. The MaNGO (Mario Negri Gynecologic Oncology) group is also a pioneering force in gynecologic oncology research and treatment. With a focus on evidence-based medicine and patient-centered care, both the MITO and the MaNGO groups play a key role in shaping standards of care and driving progress in the fight against ovarian cancer on both national and international levels.

The aim was to investigate current VTE prophylaxis practice in patients with gynecologic malignancies.

## 2. Materials and Methods

We did not need Institutional Review Board approval to publish these data since no patients were involved in this study.

We developed a survey consisting of an online self-administered questionnaire, written by G.C., reviewed and discussed by the MITO and MaNGO committees, and submitted to and approved by both the MITO and MaNGO internal review boards. 

Afterward, it was available on the MITO and MaNGO websites only to MITO-MaNGO members from 11 May 2022 to 13 June 2022.

The survey was composed of 14 multiple-choice questions, shown in Table 1. We processed one response form per center provided by the head of the center. All responses were anonymized. Descriptive analyses are detailed in the Section 3. 

The inclusion criterion was the presence of gynecologic malignancy treated in a cancer referral center. The exclusion criterion was the presence of a non-gynecologic neoplasm. 

## 3. Results

The survey was intended for centers affiliated with the MITO and MaNGO research groups. Among them, a total of 50 responses were analyzed (50% from MITO and 50% from MaNGO). 

**Figure 1:**
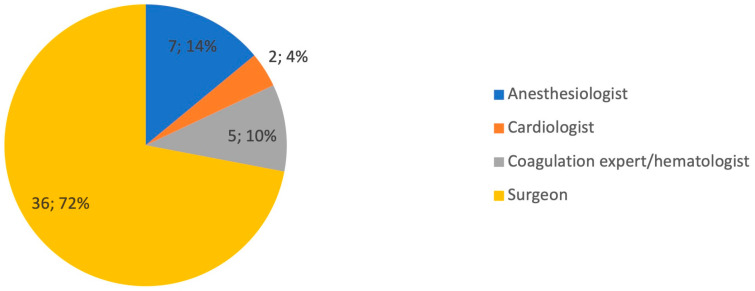
Graphical distribution of replies to question 6.

Most of the respondents were at least 40 years old (39/50, 78%) and work in a public hospital (21/50, 42%) or a university (22/40, 44%). Half of them are gynecologists (25/50, 50%) and the other half are oncologists (25/50, 50%) (Table 1). Forty-one of the respondents (82%) consider thromboembolic prophylaxis in gynecologic oncology to be relevant. In most cases (82%), there is a standardized protocol for thromboembolic prophylaxis that is used for both patients undergoing surgery and patients undergoing chemotherapy; remarkably, in 18% of cases, prophylaxis is performed exclusively in patients undergoing chemotherapy. Prophylaxis for the patient undergoing surgery is almost always managed by the surgeon (72%) (Figure 1).

**Figure 2:**
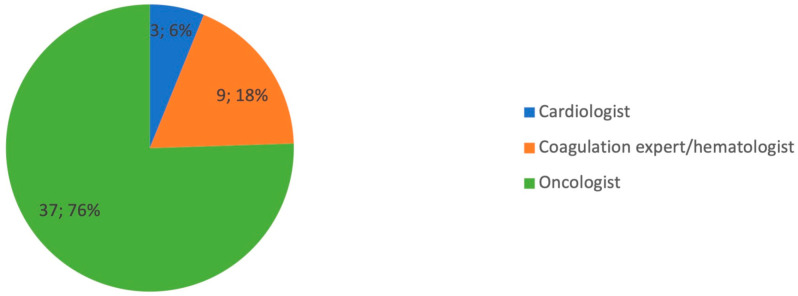
Graphical distribution of replies to question 7.

Regarding the prophylaxis of the patient undergoing chemotherapy treatment, the management is almost always carried out by the oncologist (Figure 2).

**Figure 3:**
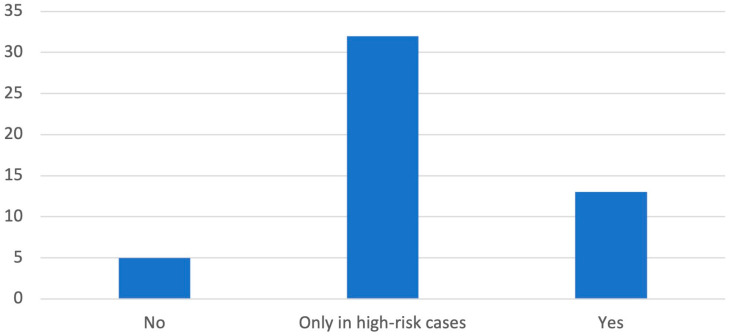
Graphical distribution of replies to question 8.

Interestingly, only 26% of respondents use a risk assessment scale—64% of those use it exclusively in high-risk patients—and in 10% of centers it is not used (Figure 3). The risk assessment scales used are the Caprini Score (6%), Khorana Score (6%), Wells Score (2%), and regional guidelines (12%). 

**Figure 4:**
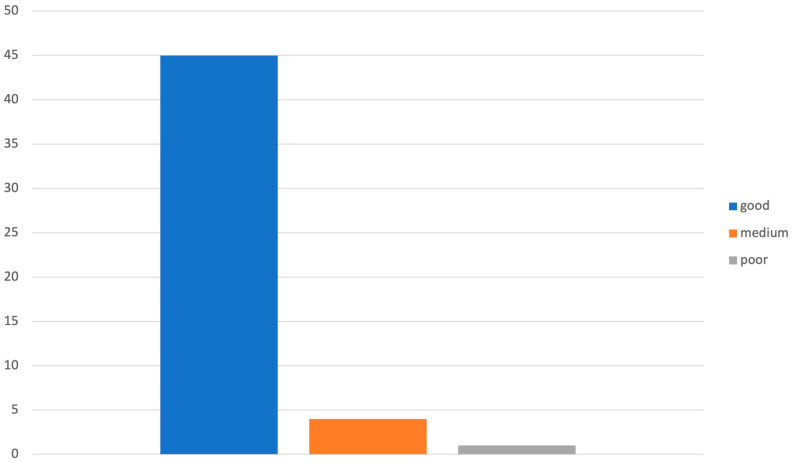
Graphical distribution of replies to question 10 with reference to LMWH level of knowledge.

**Figure 5:**
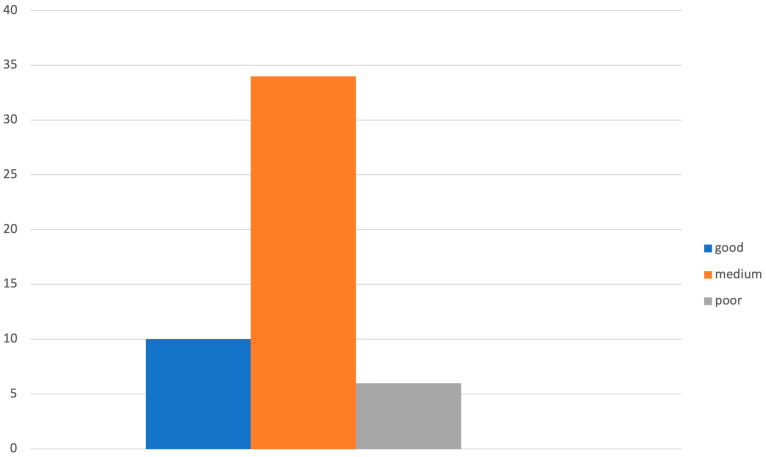
Graphical distribution of replies to question 10 with reference to DOACs level of knowledge.

Concerning drugs that can be used in thromboembolic prophylaxis, we can found that respondents have good knowledge of low-molecular-weight heparin (90%) and average knowledge of dicumarolics (40%), DOACs (68%), and antiplatelets (40%) (Figure 4 and Figure 5). 

Forty-seven respondents are aware of possible interactions between drugs used for thromboembolic prophylaxis and chemotherapy medications. Furthermore, 96% of respondents are aware of the prescribing regulations in the hospital or territory concerning drugs used for thromboembolic prophylaxis. Lastly, all interviewees consider that the MITO/MaNGO research groups should investigate the issue of thromboembolic prophylaxis in gynecologic oncology and that using a dedicated app could help manage this matter.

## 4. Discussion

This survey represents an important snapshot of the awareness and management of thromboembolic prophylaxis in gynecologic oncology in the hospitals of the MITO and MaNGO groups. Although there is high awareness among clinicians, there is still significant heterogeneity in daily clinical practice concerning thromboembolic prophylaxis protocols in cancer patients. Similar results have been obtained in past international studies, where thromboprophylaxis was underused in cancer patients at high risk of VTE. In these, only 50.6% of patients received thromboprophylaxis during their hospital stay. Moreover, cancer patients receiving cancer-specific therapy were significantly less likely to receive thromboprophylaxis than cancer patients hospitalized for other reasons [11].

In most cases, a thromboembolic risk assessment scale is only used in high-risk patients or never used at all. 

According to the latest guidelines, thromboembolic prophylaxis in gynecologic oncology should be used in the following three patient groups:

- Hospitalized medical patients;
- Hospitalized surgical patients, preoperative and postoperative;
- Ambulatory outpatients on systemic therapy.

### 4.1. Hospitalized Medical Patients

In regard to the first group of patients, we know that patients with cancer who are hospitalized are at a high risk of VTE. Even though several risk assessment models have been developed for patients admitted for medical and surgical care (for instance, the Padua Score or IMPROVE Score), none has been validated prospectively for the management of hospitalized cancer patients [12,13,14,15]. 

Prophylactic anticoagulant therapy is recommended if there are no contraindications such as active bleeding, thrombocytopenia (platelet count < 50,000/μL), underlying hemorrhagic coagulopathy (e.g., abnormal PT or aPTT that excludes a lupus inhibitor/anticoagulant) or a known bleeding disorder, or current or previous heparin-induced thrombocytopenia (HIT) (contraindication for LMWH). The recommendation is based on the assumption that ambulation in hospitalized cancer patients is not adequate to reduce the risk of VTE. Recommendations are obtained from patients with cancer hospitalized with a medical illness, most commonly acute or chronic cardiac or respiratory disease, who are hospitalized for more than 6 days, have immobility or are on bed rest for 3 or more days, are >40 years of age, and have additional risk factors for VTE [16]. Thromboprophylaxis is administered for the entire duration of the hospital stay, 6 to 14 days, or until the patient is completely ambulatory.

The latest guidelines suggest pharmacological thromboprophylaxis with LMWH over mechanical thromboprophylaxis, and pharmacological thromboprophylaxis over a combination of pharmacological and mechanical thromboprophylaxis is recommended [17]. The use of DOACs in this context, including prolonged thromboprophylaxis for 4 weeks after discharge, is not currently recommended because the reduction in VTE compared with standard heparin prophylaxis is outweighed by an increase in major bleeding [18].

A recent single-center study tried using an artificial intelligence (AI) algorithm that integrates all clinical information, laboratory data, and vital parameters of hospitalized patients and provides suggestions for the proper management of VTE prophylaxis. The VTE risk assessment scale and the bleeding risk assessment scale were also embedded in the AI algorithm. Implementing AI in managing VTE prophylaxis for cancer patients could have significant implications for healthcare. By leveraging machine learning algorithms, AI could provide personalized risk assessments, guiding clinicians in selecting appropriate prophylactic measures tailored to individual patient profiles. Moreover, AI-driven predictive models can help anticipate VTE events, enabling proactive interventions to prevent thrombotic complications. Real-time monitoring of patient data can further enhance early detection and intervention. Integrating AI into VTE prophylaxis management promises to optimize patient outcomes, reduce healthcare costs, and streamline clinical decision-making in cancer care. Despite this, no valid results were obtained and the AI needs further in-depth study so that improvements can be made [19,20].

### 4.2. Hospitalized Surgical Patients

As mentioned above, patients with a gynecologic neoplasm undergoing surgery are at a high risk of thromboembolic events. Before gynecologic surgery, routine VTE risk assessment should be performed using the Caprini Score [21,22]. Specifically, in high-risk patients, the use of perioperative prophylaxis with enoxaparin followed by extended postoperative prophylaxis, which consists of the administration of enoxaparin for 4 weeks after discharge, should be considered. Several studies have shown that extended prophylaxis results in a 55% reduction in the risk of postoperative thromboembolic events compared to patients on short-term prophylaxis. Further studies have shown that this reduction in the risk of thromboembolic events persists for three months after extended prophylaxis [17]. Direct oral anticoagulants (DOACs), such as rivaroxaban and apixaban, are accepted as an alternative treatment for cancer-associated thromboembolisms [23]. DOACs have several advantages over LMWHs, including ease of administration (orally rather than by subcutaneous injection) and unnecessary laboratory monitoring, which resulted in greater patient compliance and satisfaction overall [24]. A prospective study has shown oral apixaban is a potentially safe and less painful and easy to take alternative to subcutaneous enoxaparin for thromboprophylaxis after surgery for gynecologic cancer. Although this study did not demonstrate an increase in bleeding events during treatment, future studies are needed to confirm these findings [25].

In the case of a contraindication to anticoagulation, mechanical prophylaxis is recommended. No difference in the rate of VTE was observed in patients undergoing gynecologic oncologic surgery receiving low-dose heparin or calf intermittent pneumatic compression (IPC), even though the former was more frequently associated with postoperative bleeding complications [26]. However, IPC may not be an alternative equivalent substitute for anticoagulants in all settings. Compression stockings are a mechanical alternative prophylaxis method that could provide benefits in reducing VTE, especially when combined with other therapies [27]. Nevertheless, similar to IPC, they should not be solely relied upon as the only method of VTE prophylaxis, and both should be used when prophylactic anticoagulants are contraindicated.

### 4.3. Ambulatory Outpatients on Systemic Therapy

Chemotherapy may increase the risk of thromboembolism through at least acute damage to the vessel wall; delayed damage to vessel endothelial integrity; and through the reduction of regulatory proteins of coagulation processes, such as decreased levels of protein C and S, or decreased levels of antithrombin III (ATIII). Platinum-based therapy has been reported to induce platelet activation. Thus, gynecologic cancer patients more likely to undergo these therapies have a higher risk of VTE complications [28].

Among all ovarian cancers, the clear cell subtype is associated with the highest risk of VTE [29]. In addition to the clear cell histology, ascites is also associated with an increased risk of VTE development in patients with ovarian cancer. Furthermore, patients with stage III/IV ovarian cancer have 3.7 times the risk of VTE development compared to patients diagnosed at earlier stages. A recent systematic review evaluating the incidence of VTE during NACT in patients with ovarian cancer found that this ranges from 7.7% to 28.6%, with an overall average VTE incidence of 12.3% [30]. These patients are usually frail and have a high tumor burden and ascites, which leads to venous stasis due to compression of the mass and increased release of prothrombotic factors by the tumor cells [31].

Several guidelines do no recommend routine thromboprophylaxis because the incidence of VTE in outpatients undergoing chemotherapy was still low (1–5%) and routine thromboprophylaxis potentially exposed the patient to an increased risk of bleeding. In this setting, the Khorana risk score can be used to assess the VTE risk (excluding patients with multiple myeloma, acute leukemia, and myeloproliferative neoplasms and patients with primary/metastatic brain tumors). Those at a low risk of VTE (Khorana Score < 2) do not require routine prophylaxis. Those with an intermediate to high VTE risk score (Khorana Score ≥ 2) should receive anticoagulant prophylaxis for up to 6 months. For thromboprophylaxis beyond 6 months, an individualized approach should be considered. The drugs that may be used for VTE prophylaxis in outpatients receiving chemotherapy consist of DOACs and LMWHs. Although DOACs have gastrointestinal absorption, patients undergoing gastrointestinal tract resections may be at risk of suboptimal absorption [32].

Despite the increase in awareness of this topic, our survey revealed that thromboembolic prophylaxis is used less in outpatients undergoing systemic treatment than in those undergoing surgery. The same results can be found elsewhere; indeed, the Fundamental Research in Oncology and Thrombosis (FRONTLINE 2) survey found that only 63% of surgical oncologists used VTE prophylaxis in patients with cancer. In medically ill patients with cancer, the major reasons underlying any decision to administer prophylaxis against VTE were a prior episode of VTE (26.3% of respondents) and high-risk individuals (14.4%) [33]. Similar results were documented in the multinational ENDORSE records of patients hospitalized with medical diseases, where only 54% of patients with cancer received any form of VTE prophylaxis [34,35]. Furthermore, in a 2021 systematic review by the World Thrombosis Day steering committee, it was found that the use of adequate thromboprophylaxis still varies widely between geographical regions, from 38% in Asia to 69% in North America [34].

Our study confirmed that medical oncology must be an area from which to promote the education and use of drugs for thromboembolic prophylaxis. It also revealed how poorly VTE risk assessment scores are used. The same low use of VTE risk assessment scores as in our research groups can also be found in other international settings. In fact, in a recent American survey of oncology clinicians, 29% rarely used them and 58% did not use them at all [36]. A recent review analyzed the use of the Khorana Score in patients undergoing NACT for ovarian cancer. Specifically, it indicated that the Khorana Score’s predictive model does not adequately assess the risk of VTE in ovarian cancer patients undergoing NACT. The poor prediction of VTE when using Khorana Scores may be related to the unique symptoms of ovarian cancer. Thus, it seems that Khorana’s model is an unreliable tool for predicting VTE events in this high-risk population. Given the high incidence of VTE in this subset of ovarian cancer patients, future research should consider a new risk stratification to identify ovarian cancer patients at risk of thrombosis during neoadjuvant chemotherapy [37].

Moreover, our study found that DOACs are known and used less than LWHM. Considering the recent studies recommending their use in both the post-surgical and outpatient settings, it is advisable that there be both further studies that reinforce these findings and an adjustment of the guidelines to allow uniform clinical conduct among clinicians. 

Hence, it can be deduced how indispensable further studies are to scientifically validate the scores currently available. Indeed, the lack of validated scores represents poor adherence to VTE prophylaxis not only in gynecology but also in other disciplines such as urology [38]. 

The strength of our study consists of highlighting how the topic of thromboembolic prophylaxis is both crucial and complex. Despite guidelines, the conduct and use of VTE prophylaxis in gynecologic oncology has varied across the major centers where these malignancies are treated. Specifically, there is limited uptake in clinical practice of a risk-based approach to VTE prevention in outpatients and hospitalized cancer patients. In this context, the use of educational tools, such as an app, can easily improve the clinical management of these patients.

Our survey has a series of limitations. The most important is the possible selection bias derived from the low number of MITO and MaNGO members who participated in the questionnaire. Furthermore, the participants were exclusively gynecologists and oncologists, and as we observed, in some Italian hospitals, thromboembolic prophylaxis is managed by the cardiologist, anesthesiologist, or coagulation/hematology expert, so the omission of these specialists could represent another potential bias. Nevertheless, the survey focused on critical issues in our daily clinical management of oncologic patients such as the under-evaluation and under-prescription of thromboembolic prophylaxis in the oncologic outpatient setting, indicating that urgent measures of education and awareness among clinicians should be taken. In this context, the use of easy and manageable tools created and distributed to our colleagues, such as a dedicated app containing all the scores and available guidelines for thromboembolic prophylaxis, may improve the clinical management of gynecologic oncologic patients.

## Figures and Tables

**Table 1:** Full list of questions and answers.

Question	Answer	Percentage
1. Age		
<40 years old	11	22%
>40 years old	39	78%
2. Specialty		
Gynecologist	25	50%
Oncologist	25	50%
3. Structure		
Private hospital	5	10%
Public hospital	21	42%
University hospital	22	44%
Outpatient setting	2	4%
4. How relevant do you perceive thromboembolic prophylaxis in gynecologic oncology?		
Irrelevant	1	2%
Averagely relevant	8	16%
Highly relevant	41	83%
5. The center where you work has a standardized protocol for the management of thromboembolic prophylaxis in gynecologic oncology		
No	0	0%
Only for surgical prophylaxis	22	44%
Only for prophylaxis to patients undergoing chemotherapy	9	18%
For all patients	19	38%
6. Who manages thromboembolic prophylaxis in patient undergoing gynecologic oncology surgery?		
Surgeon	36	72%
Anesthesiologist	7	14%
Cardiologist	2	4%
Coagulation expert/hematologist	5	10%
7. Who manages thromboembolic prophylaxis in patient undergoing chemotherapy for gynecologic malignancy?		
Oncologist	37	74%
Cardiologist	3	6%
Coagulation expert/hematologist	10	20%
8. Do you use a thromboembolic risk assessment scale?		
Yes	13	26%
No	5	10%
Only in high-risk cases	32	64%
9. If the answer is yes, which evaluation system scale do you prefer?		
Caprini Score	3	6%
Khorana Score	3	6%
Wells Score	1	2%
Regional guidelines	6	12%
10. State your level of knowledge of the following categories of drugs that may be used for thromboembolic prophylaxis		
Direct oral anticoagulants (DOACs)		
Poor	6	12%
Medium	34	68%
Good	10	20%
Low-molecular-weight heparin		
Poor	1	2%
Medium	4	8%
Good	45	90%
Antiplatelets		
Poor	3	6%
Medium	20	40%
Good	27	54%
Dicumarolics		
Poor	15	30%
Medium	20	40%
Good	15	30%
11. Are you aware of any interactions between drugs used for thromboembolism prophylaxis and antiblastic drugs?		
Yes	30	60%
No	3	6%
Slightly	17	34%
12. Are you aware of hospital and territory prescribing regulations related to thromboembolic prophylaxis drugs?		
Yes	34	68%
No	2	4%
Slightly	14	28%
13. Do you think the MITO/MaNGO research groups should further investigate this topic?		
Yes	50	100%
No	0	0%
14. Do you think a designated app can help you manage this matter?		
Yes	49	98%
No	1	2%

## Data Availability

The data presented in this study are available upon reasonable request from the corresponding author.
